# Dynamic morphometric characterization of local connective tissue network structure in humans using ultrasound

**DOI:** 10.1186/1752-0509-1-25

**Published:** 2007-06-05

**Authors:** Helene M Langevin, Donna M Rizzo, James R Fox, Gary J Badger, Junru Wu, Elisa E Konofagou, Debbie Stevens-Tuttle, Nicole A Bouffard, Martin H Krag

**Affiliations:** 1 Department of Neurology, University of Vermont, Burlington, VT, USA; 2 Department of Civil & Environmental Engineering, University of Vermont, Burlington, VT, USA; 3 Department of Orthopaedics & Rehabilitation, University of Vermont, Burlington, VT, USA; 4 Department of Medical Biostatistics, University of Vermont, Burlington, VT, USA; 5 Department of Physics, University of Vermont, Burlington, VT, USA; 6 Department of Biomedical Engineering, Columbia University, New York, NY, USA

## Abstract

**Background:**

In humans, connective tissue forms a complex, interconnected network throughout the body that may have mechanosensory, regulatory and signaling functions. Understanding these potentially important phenomena requires non-invasive measurements of collagen network structure that can be performed in live animals or humans. The goal of this study was to show that ultrasound can be used to quantify dynamic changes in local connective tissue structure *in vivo*. We first performed combined ultrasound and histology examinations of the same tissue in two subjects undergoing surgery: in one subject, we examined the relationship of ultrasound to histological images in three dimensions; in the other, we examined the effect of a localized tissue perturbation using a previously developed robotic acupuncture needling technique. In ten additional non-surgical subjects, we quantified changes in tissue spatial organization over time during needle rotation vs. no rotation using ultrasound and semi-variogram analyses.

**Results:**

3-D renditions of ultrasound images showed longitudinal echogenic sheets that matched with collagenous sheets seen in histological preparations. Rank correlations between serial 2-D ultrasound and corresponding histology images resulted in high positive correlations for semi-variogram ranges computed parallel (r = 0.79, p < 0.001) and perpendicular (r = 0.63, p < 0.001) to the surface of the skin, indicating concordance in spatial structure between the two data sets. Needle rotation caused tissue displacement in the area surrounding the needle that was mapped spatially with ultrasound elastography and corresponded to collagen bundles winding around the needle on histological sections. In semi-variograms computed for each ultrasound frame, there was a greater change in the area under the semi-variogram curve across successive frames during needle rotation compared with no rotation. The direction of this change was heterogeneous across subjects. The frame-to-frame variability was 10-fold (p < 0.001) greater with rotation than with no rotation indicating changes in tissue structure during rotation.

**Conclusion:**

The combination of ultrasound and semi-variogram analyses allows quantitative assessment of dynamic changes in the structure of human connective tissue *in vivo*.

## Background

Connective tissue, including the fasciae surrounding all muscles and organs, forms a continuous network throughout the body. This network also includes superficial subcutaneous connective tissue (pannicular fascia) which forms interconnecting longitudinal fibrous sheets intermixed with adipose tissue and linked to the skin by string-like "skin ligaments" [1-9]. Recent evidence suggests that this body-wide connective tissue network is not only composed of a web-like collagenous matrix, but also includes a network of fibroblasts that are dynamically responsive to mechanical stimulation [10-13]. These fibroblasts are linked to the collagen matrix by specialized proteins, as well as to each other by abundant cell-to-cell contacts [10,14]. Modern understanding of complex systems suggests that, given these two types of interconnections, the dynamic responsiveness of fibroblasts within connective tissue may be affected by the structural characteristics of the collagenous network both locally and at a distance [15,16]. Understanding these potentially important phenomena requires non-invasive measurements of both collagen network structure and cell function that can be performed in live animals or humans. Such measurements could be used to quantify changes over time and in response to specific experimental stimuli. Recently developed techniques using injectable fluorescent tagged proteins allow functional assessment of cells in live animals [17]. Non-invasive *in vivo *measurement of collagen network microstructure has been previously achieved using polarization sensitive optical coherence tomography (PS-OCT) [18,19]. However, this technique can only visualize structures within 1–2 millimeters of the skin surface, and is not well suited to the examination of deeper subcutaneous and perimuscular connective tissues. A technique allowing quantitative evaluation of both superficial and deep connective tissue network structure therefore may open up a new area of investigation in systems biology.

For many organ systems (e.g. cardiovascular), ultrasound is increasingly used to visualize human anatomy non-invasively and obtain *in vivo *tissue measurements [20-24]. In addition, elasticity imaging techniques use signal processing methods on consecutive ultrasound frames to detect tissue motion and strain, providing biomechanical as well as morphological information from ultrasound data [25]. Elastography also has had an important impact on several clinical and research applications, such as the detection of differences in tissue stiffness associated with pathology in the breast [26,27], prostate [28,29], blood vessels [30], heart [31], skin [32]and cartilage [33]. During ultrasound imaging of biological materials, echoes generated by homogeneous material (e.g. fat) produce diffusely scattered signals, while echoes generated by interfaces of organized tissues with different acoustic impedances (product of density and speed of sound) produce more correlated "specular" signals [34-37]. It is generally assumed that the echogenic bands seen in human subcutaneous tissue correspond to connective tissue. This notion is supported by studies of connective tissue structures using both ultrasound and magnetic resonance imaging (MRI) [21,38], as well as the successful use of echogenic bands as landmarks during ultrasound-guided percutaneous procedures such as regional anesthesia [39,40]. However, to our knowledge, the correspondence between connective tissue structures revealed by ultrasound and "gold-standard" histological tissue identification has not been demonstrated quantitatively. Since human connective tissue contains a combination of adipose and fibrous (collagenous) tissues, the majority of specular echoes forming ultrasound images of connective tissue are expected to originate from interfaces between regions with predominantly fat (density = 0.917–0.940 g/cm^3^, speed of sound = 1,410–1,480 m/s), regions with predominantly collagen (density = 1.1–1.2 g/cm^3^, speed of sound = 1,540–1,570 m/s), and regions with predominantly glycosaminoglycans and water (density 0.998–1.05 g/cm^3^, speed of sound = 1480–1,500 m/s). Several parameters, including the ratio of specular-to-diffuse backscatter coefficients and the ultrasound intensity signal-to-noise ratio, have been used to assess tissue organization by quantifying the presence of organizational scatterers within tissues [35,41-45]. These measures, however, do not reflect structural continuity in a given plane or direction, which is one of principal characteristics of connective tissue. Semi-variogram (also termed "variogram") analysis is a technique so far little applied to biology but commonly used in the hydrogeological and geophysical communities to evaluate the organization of laminar structures beneath the earth's surface [46-50]. Statistical analysis of ultrasound images using semi-variograms therefore offers a new way to quantify the organizational pattern of connective tissue *in vivo*, specifically regarding the structural continuity of longitudinal collagenous sheets, as well as changes in this structural organization over time.

The goals of this study were 1) examine the degree of correspondence between the three dimensional pattern of echoes generated by ultrasound imaging of connective tissue and the three dimensional pattern formed by fibrous connective tissue in histological serial sections of the same piece of tissue and 2) show that ultrasound can be used to quantify dynamic changes in connective tissue structure *in vivo *induced using a previously developed robotic acupuncture needling technique [51,52]. We have previously demonstrated in animal experiments that rotation of an inserted ultra-fine acupuncture needle will cause winding and gathering of collagen fibers from the periphery toward the needle, and therefore can be used to achieve highly specific mechanical perturbations of fibrous connective tissue [52-54]. In this study, we have combined robotic needling with ultrasound elasticity imaging techniques, ultrasound semi-variogram analyses and histology to quantify structural changes occurring in connective tissue as a result of needle rotation. We used 1) elasticity imaging techniques to map areas of tissue motion, 2) histology to identify tissue structures in relation to ultrasound images and 3) semi-variograms to quantify changes in tissue spatial organization over time.

## Results and discussion

### Comparison of three dimensional ultrasound and histology image visualizations

**Figure 1:**
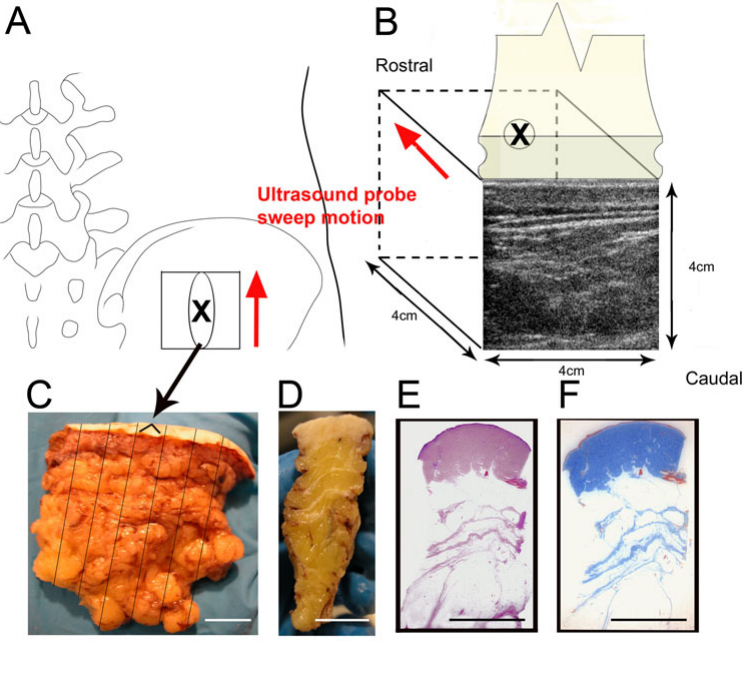
Methods used for ultrasound and histology image acquisition in a human subject undergoing surgery. A,B: location and size of ultrasound scan area on the back (X indicates the center of the scanned area in both A and B); C: excised tissue sample indicating location of seven serial tissue blocks; D,E,F: fixed tissue block cut transversely with corresponding hematoxylin/eosin (E) and Masson trichrome (F) histological slides. Scale bars, 1 cm.

**Figure 2:**
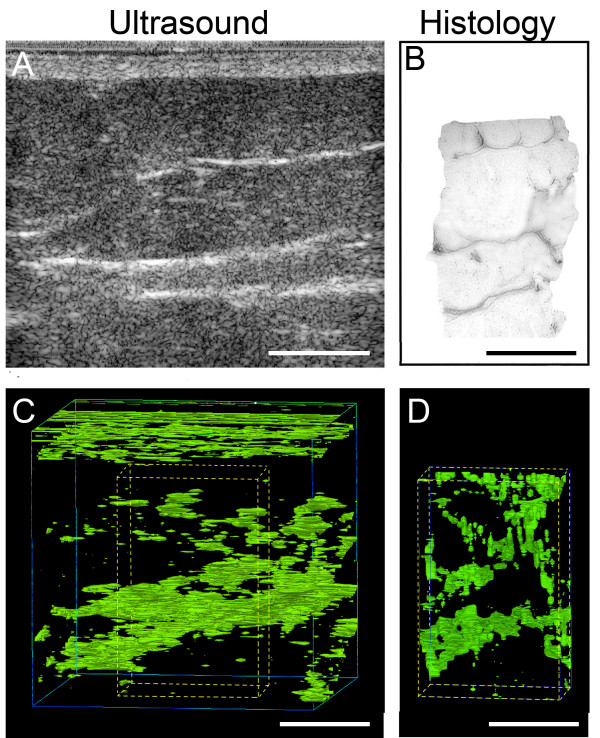
Ultrasound and histology images in a human subject undergoing surgery (ultrasound images and biopsy taken at the same location on the back). A,B: Sample single ultrasound and corresponding digitized (grey scale) histology section of human subcutaneous tissue. C,D: Three-dimensional isocontour visualizations based on ultrasound (C) and histology (D) data. Dashed lines in C represent the 3-d location of histology block shown in D. Scale bars, 1 cm.

In one anesthetized subject undergoing spinal fusion surgery, we first performed ultrasound examination of a 4 × 4 × 4 cm^3 ^cube of tissue at the bone graft site, then excised a corresponding subcutaneous tissue biopsy specimen that was fixed and processed for histology (Figure 1), (see methods section at end of paper for details). Our goal was to compare the three dimensional pattern formed by echoes in the ultrasound images with the three dimensional pattern formed by fibrous connective tissue in the same piece of tissue. Distinct transverse echogenic bands in the 2-d ultrasound images (Figure 2A) visually matched with collagenous bands on the corresponding histology images (Figure 2B). Computerized visualizations of serial ultrasound (Figure 2C) and histology (Figure 2D) images showed similar longitudinal "sheet-like" structures with both methods (see Additional file 1 showing 3-d animated rendition of collagenous sheets).

**Figure 3:**
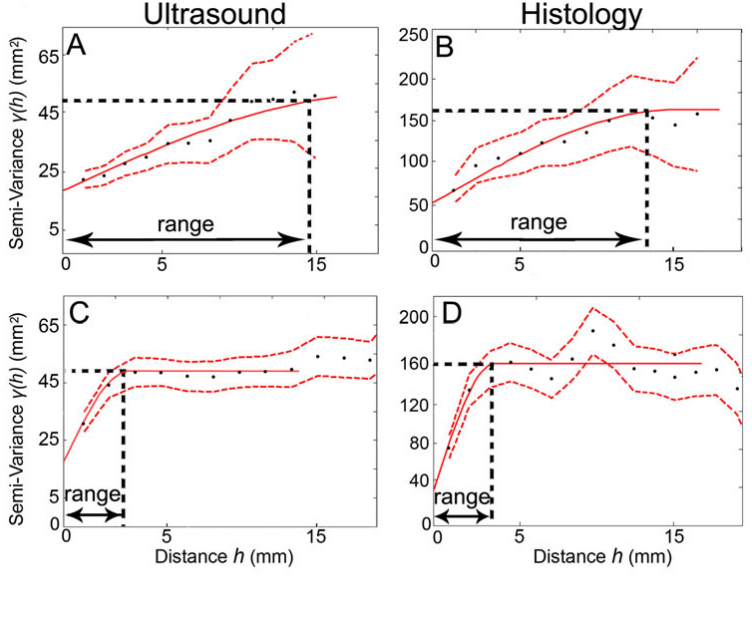
A, B: Example of semi-variogram plots calculated in the direction parallel to the skin from one matched pair of ultrasound (A) and histology (B) images (corresponding to the single ultrasound and histology images shown in Figure 2 A, B). C, D: Semi-variogram plots for the same two images calculated in the direction perpendicular to the skin. *γ(h) *is the semi-variance of pixel intensity for pairs of pixels separated by a given distance *(h) *(see methods). Solid red lines represent fitted experimental semi-variograms. Dashed red lines represent 95% confidence limits.

Figure 3 shows semi-variograms plots calculated from one matched pair of ultrasound (A,C) and histology (B,D) images corresponding to the single ultrasound and histology images shown in Figure 2A, B. The shapes (best fit models) of the ultrasound and histology semi-variogram curves for matching ultrasound and histology images were similar to each other in both parallel (Figure 3A, B) and perpendicular (Figure 3C, D) directions relative to the skin surface. The example shown in Figure 3 also illustrates that, with both ultrasound and histology, the semi-variogram ranges were in general greater in the parallel than the perpendicular direction, (a larger semi-variogram range in a given direction indicates spatial continuity over a greater distance in that direction). The rank correlation for the semi-variogram range between the 23 serial ultrasound and corresponding histology images was r = 0.79, P < 0.001 for the direction parallel to the skin. For the perpendicular direction, the rank correlation was r = 0.63, P < 0.001. Although this comparison of ultrasound and histology was limited to one subject, the high concordance found between these ultrasound and histology data sets suggests that 1) the similarity in spatial structure between the ultrasound and histology data sets was not due to chance and 2) semi-variogram analyses of ultrasound images can be a useful tool to quantify the spatial structure of connective tissue *in vivo*.

### Dynamic changes in connective tissue structure during needle rotation

**Figure 4:**
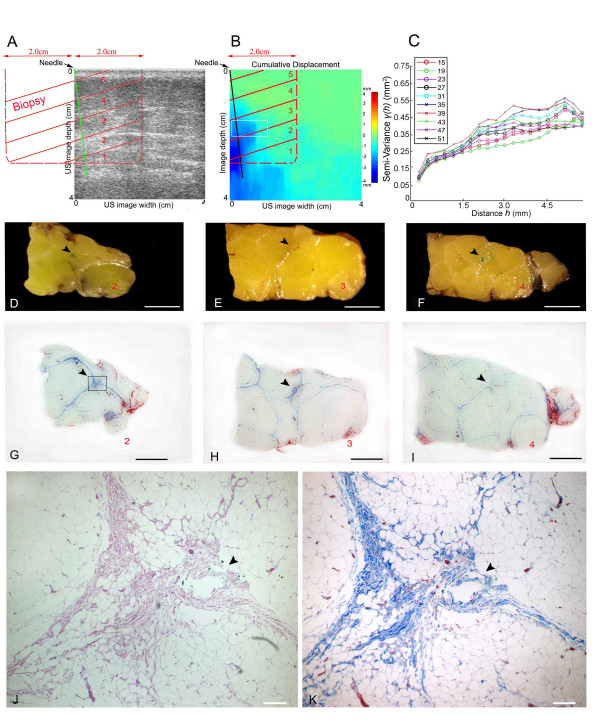
Effect of needle rotation on connective tissue using ultrasound and histology in a human subject undergoing acupuncture needling on the back during surgery. A: relative locations of ultrasound image, needle and biopsy blocks (labelled 1–5, red letters). B: tissue displacement map during needle rotation shown with ultrasound elasticity imaging showing maximal displacement in the area of the needle. C: semi-variogram analysis of sequential ultrasound frames during needle rotation. Semi-variograms were computed from all ultrasound data points included in the region of interest (10 mm × 5 mm) indicated by white box on panel B. Each semi-variogram line represents one ultrasound frame (frame number shown in inset). D-I: gross (D-F) and microscopic (G-I) images of tissue sections 2,3 and 4 (see location in panels A,B). Arrowheads indicate location of needle (marked with green ink in the tissue specimen). J, K: higher magnification of box area in (G) stained with Hematoxylin/Eosin (J) and Masson Trichrome which stains collagen blue (K). Scale bars: 1 cm (C-I), 0.2 mm (J-K).

We first examined the effect of acupuncture needle rotation in one subject undergoing surgery using a combination of ultrasound and histology. We acquired ultrasound images continuously during robotic acupuncture needle rotation at the bone graft site, then excised the corresponding piece of tissue with the needle still in place. Figure 4A shows the relative locations of ultrasound images, biopsy and needle. In Figure 4B, a spatial plot of cumulative tissue displacement during needle rotation (calculated using cross correlation techniques) shows that tissue displacement was maximal in the area surrounding the needle. Figure 4D–K show that, in histological sections, a "whorl" of connective tissue was present in the area surrounding the needle corresponding to the area of maximal tissue displacement in Figure 4B. This connective tissue whorl was similar to that observed in previous animal studies [53,54]. At the same location surrounding the needle, semi-variogram analysis of B-scan ultrasound images revealed a gradual change in the shape of the semi-variogram curve during the successive ultrasound frames acquired during needle rotation (Figure 4C). This suggested that needle rotation caused not only tissue movement, but also change in tissue structure.

**Figure 5:**
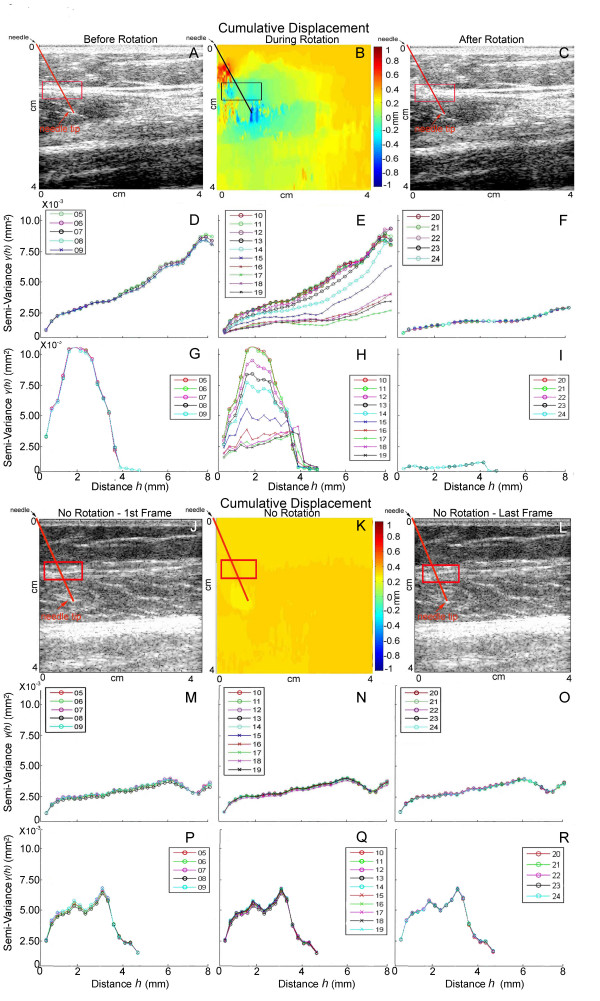
Effect of needle rotation (A-I) compared with no rotation (J-R) in a non-surgical subject needled on the thigh bilaterally shown with ultrasound B-scans (A,C,J,L), tissue displacement maps (B,K) and semi-variogram plots (D-I,M-R). Needle position is indicated by oblique line originating from left upper corner of images. Tissue displacement maps show maximal tissue displacement in the area surrounding the needle with rotation (B) compared with no rotation (K). Semi-variograms were computed from all ultrasound data points included in the region of interest (10 mm × 5 mm) indicated by box in the directions parallel (D-F, M-O) and perpendicular (G-I, P-R) to the skin. In Panels D-I, each semi-variogram line represents one ultrasound frame before rotation (D,G), during rotation (E,H) and after 8 full uni-directional rotations (F,I). Panels M-R show semi-variograms for sequential frames with needle insertion alone without rotation (time period equivalent to panels D-I). Frame numbers are shown in inset for each semi-variogram.

**Figure 6:**
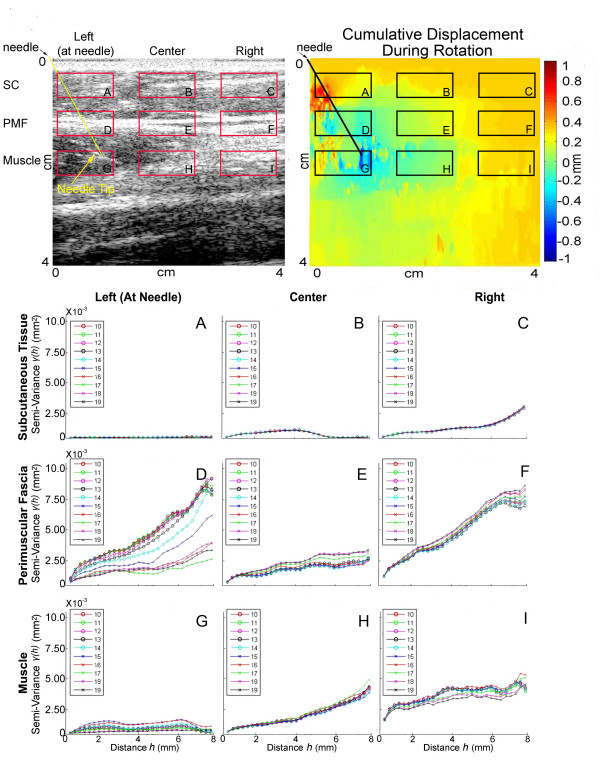
Semi-variogram plots during needle rotation in the same non-surgical subject shown in Figure 5. Semi-variograms correspond to different regions of interest (all 10 mm × 5 mm) shown on B-scan image (top left) and cumulative displacement map (top right). Regions of interest A-C are located within subcutaneous tissue (SC), regions D-F are within perimuscular fascia (PMF) and regions G-I are within muscle. Needle position is indicated by oblique line originating from left upper corner of the B-scan and displacement images.

**Figure 7:**
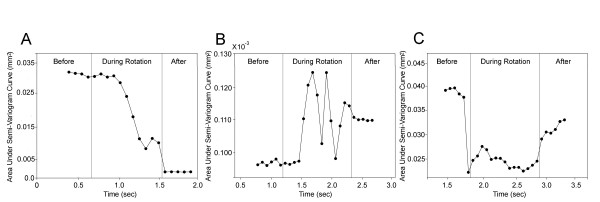
Area under the semi-variogram curve (AUC) plotted over successive ultrasound frames before, during and after needle rotation. Panels A-C correspond to three different non-surgical human subjects needled on the thigh.

In order to further investigate whether needle rotation consistently induces change in tissue structure that is measurable with ultrasound, we tested ten non-surgical subjects. In each subject, we acquired ultrasound images during needling at the same point on the thigh bilaterally, with right and left sides of the body randomized to needle rotation vs. no rotation. Figure 5 shows ultrasound B-scans, cumulative tissue displacement and semi-variograms in a representative subject during needle rotation (Figure 5A–I) compared with no rotation (Figure 5J–R). Figure 5B shows that cumulative tissue displacement during rotation was greatest in the area surrounding the needle. Comparison of semi-variograms computed for successive ultrasound frames with and without rotation revealed that a greater change in the shape of the semi-variogram curve occurred during rotation (Figures 5E, H), compared with no rotation (Figure 5N, Q). The greatest effects of rotation were seen at the intersection of the needle with the perimuscular connective tissue plane, indicating a specific effect of the needle on connective tissue, rather than non-specific movement of the entire tissue region (Figure 6). In some subjects, the area under the semi-variogram curve (AUC) increased during rotation, while it decreased in others (Figure 7). The change in AUC in some cases persisted during the post-rotation period, (Figure 7A, B) suggesting that plastic, rather than transient, tissue deformation had occurred. In other cases, the tissue appeared to "rebound" toward its original conformation after the end of rotation (Figure 7C). Consistent with this heterogeneous response across subjects, we found no evidence of differences in the mean AUC (averaged within each subject over all ultrasound frames) between rotation and no rotation conditions among the ten subjects tested (repeated measures ANOVA p = 0.69). Rotation did, however, produce a significant increase in frame-to-frame variability: there was a greater than 10-fold increase (p < 0.001) in the variability of the area under the semi-variogram curve (V_AUC_) during rotation compared with no rotation among the ten subjects. For semi-variograms calculated parallel to the skin, V_AUC _was 0.0686 during rotation compared with 0.0050 no rotation. Similar results were obtained in the perpendicular direction (V_AUC _was 0.0228 vs. 0.0012 for rotation and no rotation respectively). When the order of frames was taken into consideration, the mean absolute change in area under the semi-variogram curve (AUC) across successive frames was approximately two-fold greater during rotation compared to no-rotation both parallel (0.070 vs. 0.035, p=.007) and perpendicular (0.048 vs. 0.022, p=.03) to the skin.

Together, our results show that 1) needle rotation produced changes in connective tissue structure and 2) individuals responded differently from one another in response to the same mechanical input (i.e. needle rotation). This heterogeneous response of tissues observed among subjects was interesting and may be due to differences in initial tissue structure. Increased or decreased structural continuity over time may correspond to collagenous bundles either becoming more straight and tightly organized (i.e. less wavy) or "breaking up" due to the mechanical pull resulting from the tissue winding around the needle. Whether collagen bundles straighten or break in response to a given mechanical input may depend on a number of factors including collagen bundle size, cross-linking, tissue glycosaminoglycan concentration and water content [55-57]. Further studies will be needed to examine dynamic tissue behavior in relation to initial tissue factors and may lead to important new insights on how connective tissue behaves in the presence of pathology including injury, inflammation, scarring and fibrosis.

A potential limitation of structural tissue analysis based on ultrasound imaging is that the strength of reflected ultrasound signals is dependent to some degree on the orientation of a given structure relative to the direction of the incident wave. In this study, because the ultrasound transducer was placed perpendicular to the skin, organized structures oriented parallel to the skin would be expected to generate stronger echoes than structures perpendicular to the skin. Figure 2 indeed suggests that the small transverse septae within subcutaneous tissue are less well seen in the ultrasound images compared with the large longitudinal sheets. However, the close similarity between ultrasound and histology semi-variograms for both directions (parallel and perpendicular to the skin) indicates that ultrasound, nevertheless, captured the dominant structural characteristics of the tissue. Further studies examining other types of connective tissue should consider this limitation and control for ultrasound transducer position when comparing different experimental conditions.

## Conclusion

The complementary techniques used in this study show that the laminar collagenous structures seen with ultrasound reorganized during rotation of an inserted needle. These changes were localized to the area of the needle and were accompanied by a "whorl" of collagen in corresponding biopsy sections. The combination of ultrasound and semi-variogram analysis used in this study is novel in biology. These imaging methods may be used along with dynamic visualization of molecular markers and computational modeling to investigate connective tissue as a functional network system capable of responding to local perturbations.

## Methods

Twelve human subjects (11 females, 1 male; age range 24–74) were recruited and signed informed consent for this study. The Institutional Review Board at the University of Vermont approved the study methods and procedures. Two subjects were patients undergoing spinal fusion surgery (one 74 year old female and one 47 year old female). In the first surgical subject, we examined the degree of correspondence between serial ultrasound and histology images of the same tissue sample. In the second surgical subject, we examined structural changes occurring during robotic needle rotation using both ultrasound and histology, (ultrasound data was acquired with the transducer in a fixed position while an inserted needle was rotated, followed by tissue excision with the needle still in place). In the ten non-surgical subjects, we performed robotic acupuncture needling at the same anatomical location on the thigh bilaterally to compare the effect of needle rotation vs. needle insertion alone without rotation.

### Comparison of ultrasound and histology three dimensional image visualizations (N = 1 subject)

With the patient anesthetized, a 4 cm × 4 cm skin surface area located at the bone graft site, (centered on an area 4 cm lateral to the posterior superior iliac spine and 4 cm caudal to the iliac crest) was imaged by ultrasound (Figure 1A, B). An ultrasound cine-recording was acquired using a GE System Five (Vingmed) scanner equipped with a linear array ultrasound transducer (frequency 10 MHz). The transducer was translated across the defined area at a rate of 2.5 mm/second while acquiring ultrasound images at 10 frames/second with an ultrasound image depth of 4 cm, yielding serial 3-d data on a 64 cm^3 ^cube of tissue with pixel intensity ranging from 0–255. After ultrasound imaging, a 4 cm × 2 cm × 4 cm tissue sample was excised at the site of the surgical incision, centered on the middle of the area scanned by ultrasound (Figure 1A, C). The tissue was cauterized at the rostral end for orientation purposes, and then fixed in 10% formalin. After fixation the tissue sample was cut transversely into seven 4 mm thick blocks (Figure 1C, D). The seven tissue blocks were paraffin-embedded and serial-sectioned at 10 μm thickness. Every 25th section was stained with hematoxylin/eosin (H&E). Sections were photographed using an Olympus Bx51 reflected light microscope (Melville, NY). The H&E images were digitized and converted to gray scale. A smaller number of additional serial sections also were stained with Masson Trichrome, and compared with the corresponding H&E images to verify the accuracy of H&E staining in defining the outline of the collagenous subcutaneous connective tissue structures (Figure 1E, F). In order to assess changes in tissue dimensions during processing, we measured the dimensions of 1) the freshly excised whole tissue biopsy, 2) the whole fixed biopsy, 3) each trimmed tissue block before paraffin embedding 4) paraffin embedded tissue blocks and 5) tissue sections on histology slides. We found that minimal tissue shrinkage occurred during this overall process (on average, tissue measurements increased by 6.1% after paraformaldehyde fixation and decreased by 6.8% after paraffin embedding). We therefore did not include a shrinkage factor in the 3-d rendition of histological images.

The ultrasound cine-recording included 160 individual 2-D frames. Each ultrasound frame was cropped to match the area of the histology image. Histology slides were stacked sequentially using major landmarks of the tissue (e.g. blood vessels) for orientation. However, tissue landmarks were not used to match ultrasound and histology images with each other. Rather, ultrasound and histology data sets were matched as follows: the histology section corresponding to the middle of the biopsy (middle histology section) was first identified based on serial section numbers; this histology section was then matched with the ultrasound image corresponding to the middle of the ultrasound recording (80^th ^frame); next, x coordinates were matched by lining up the medial-lateral midpoint of the middle histology section with the medial-lateral midpoint of the ultrasound image; finally, y coordinates were matched by lining up the deep border of the dermis in the middle histology section to the deep border of the dermis in the middle ultrasound image. Thus, histology sections were stacked relative to each other before matching the whole histology data set with the serial ultrasound images. This avoided the potential bias associated with using connective tissue layers themselves as landmarks. Three-dimensional visualizations of ultrasound and histology image stacks were performed using Environmental Visualization EVS-Pro V (8.0) software.

### Effect of needle rotation using ultrasound and histology (N = 1 subject)

With the patient anesthetized, an ultrasound cine-recording was acquired at the same location as above, with the transducer in a fixed position while an inserted needle was rotated, followed by tissue excision with the needle still in place. Needle insertion and rotation were performed using a computer-controlled needling instrument as previously described [51,52] and disposable stainless steel acupuncture needles (Seirin, Japan, 0.25 mm in diameter and 50 mm in length). The needling instrument and ultrasound transducer were placed in a clamp system such that the transducer was perpendicular to the skin and the needling instrument was at a 20° angle with respect to the transducer. Needle insertion depth was 40 mm. Needle rotation and axial translational speeds were 6 revolutions/sec and 10 mm/s respectively. The needle was rotated for 4 seconds for a total of 24 continuous uni-directional revolutions.

An ultrasound recording consisting of seventy successive frames was acquired continuously during the needling procedure at 13.2 frames/s. Ultrasound frequency was 6.9 MHz. Raw ultrasonic radio frequency (RF) data were collected and stored following digitization by the ultrasound system at a sampling frequency of 20 MHz.

After ultrasound data acquisition, the needling instrument was disconnected from the needle, leaving the needle in place within the tissue. A 4 cm × 2 cm × 4 cm tissue sample was then excised as above, centered on the needle. After fixation, the position of the needle in the tissue was marked by dipping the tip of the needle (protruding from the end of the tissue) into green ink before pulling it out of the tissue, thus dragging the ink through the tissue as the needle was being pulled back. The tissue sample was then cut into five blocks perpendicular to the needle as shown in Figure 4A, D, E, F and processed for histology.

### Comparison of needle rotation vs. needle insertion without rotation (N = 10 subjects)

Ultrasound cine-recordings were acquired at 13.2 frames/sec as described above on the anterior thigh (18 cm superior to the middle of the superior edge of the patella) bilaterally. The two testing locations were randomized to needle rotation (8 clockwise uni-directional revolutions) vs. no rotation. Needle insertion depth was calculated for each individual so that the needle tip extended 5 mm past the peri-muscular fascia, and the needle insertion angle was 45° to the transducer. Both raw radio-frequency (RF) ultrasound data and B-scan image were saved for quantification of tissue displacement (elasticity imaging) and changes in tissue structure (semi-variogram analysis).

### Ultrasound data post-processing for measurement of tissue displacement during needle rotation (using elasticity imaging techniques)

The displacement occurring between each of the seventy successive frames acquired during needle rotation was calculated using cross-correlation techniques with a 3 mm window and a window overlap of 90% as previously described [25,52]. The term 'displacement' refers to the incremental axial (i.e. along the propagation of the ultrasound beam, or top to bottom, on all images shown) motion that the tissue undergoes between two successively acquired ultrasound frames (i.e. after 76 ms have elapsed).

### Semi-variogram analyses

Semi-variogram analyses statistically evaluate the spatial structure (i.e. the general form, magnitude and spatial scale of the variation) in a particular data set using a semi-variogram plot. In this study, we have computed the semi-variance, *γ*(*h*), defined as the spatial dissimilarity in intensity between image pixels separated by a range of distances, *h*, in a chosen direction:

γ(h)=12N(h)∑[u(a)−u(a+h)]2,
 MathType@MTEF@5@5@+=feaafiart1ev1aaatCvAUfKttLearuWrP9MDH5MBPbIqV92AaeXatLxBI9gBaebbnrfifHhDYfgasaacH8akY=wiFfYdH8Gipec8Eeeu0xXdbba9frFj0=OqFfea0dXdd9vqai=hGuQ8kuc9pgc9s8qqaq=dirpe0xb9q8qiLsFr0=vr0=vr0dc8meaabaqaciaacaGaaeqabaqabeGadaaakeaaiiGacqWFZoWzcqGGOaakcqWGObaAcqGGPaqkcqGH9aqpdaWcaaqaaiabigdaXaqaaiabikdaYiabd6eaojabcIcaOiabdIgaOjabcMcaPaaadaaeabqaaiabcUfaBjabdwha1jabcIcaOiabdggaHjabcMcaPiabgkHiTiabdwha1jabcIcaOiabdggaHjabgUcaRiabdIgaOjabcMcaPiabc2faDnaaCaaaleqabaGaeGOmaidaaaqabeqaniabggHiLdGccqGGSaalaaa@4B27@

where *N(h) *is the number of pixel pairs separated by the distance, *h*; and *u(a) *and *u(a*+*h) *are the respective pixel intensities at locations (*a*) and some distance away (*a+h*). The difference in pixel intensity between each data point and every other data point in a given direction was calculated and these differences were binned according to separation distance *(h) *(see horizontal axes in Figure 3). For example, all pairs of points separated by a distance ranging between 0 and 10 pixels were included in the first bin and represented by the first data point of Figure 3; the y axis represents the average semi-variance in pixel intensity associated with these pairs of data. Semi-variance values were calculated and averaged over each bin using MATLAB version 6.1 (MathWorks, Inc, Natick, MA).

In this study, we used semi-variograms to 1) compare B-scan ultrasound images with histology based on quantitative measures representing tissue structure and 2) quantify changes in tissue structure over time during needle rotation. For the ultrasound/histology comparison, we used a standard semi-variogram parameter (the range) derived using non-linear regression models fit using JMP statistical software (SAS Institute Inc., Cary, NC) [58-60]. The range is the most commonly used measure of spatial structure for spatially auto-correlated data sets and is defined as the distance at which *γ(h) *(the variability in the difference in pixel intensity between pairs of points) reaches a plateau. Larger ranges correspond to less spatial variability and therefore greater spatial correlation (*i.e*. points located large distances apart are still correlated) while smaller ranges imply more spatial variation and therefore weaker spatial correlation. Spearmans's rank correlation was used to examine the concordance between estimates obtained from ultrasound and histology.

The area under the semi-variogram curve (AUC) was used to examine structural changes in tissue during needle rotation. The AUC was calculated using the trapezoidal rule from the raw semi-variogram data corresponding to each B-scan ultrasound image acquired during needle rotation (or no rotation). We used the AUC as a surrogate measure for the range as it provides a quantitative measure of spatial structure without necessitating non-linear curve fitting for each of the 300 ultrasound frames (15 frames × 2conditions × 10 subjects) in parallel and perpendicular directions. The AUC is sensitive to change in data structure, and was found to be highly correlated with the range in the ultrasound/histology comparison (r = 0.88, P < 0.001 and r = 0.90, P < 0.001 for directions parallel and perpendicular to the skin respectively).

Repeated measures analysis of variance was used to compare mean AUC between rotation and no-rotation conditions. The mean AUC for each condition was based on the average of the 15 frames across the 10 subjects. Analysis of variance was also used to compare rotation and no-rotation conditions with respect to the mean absolute difference in AUC between successive frames. This outcome measure examines structural changes while accounting for the temporal ordering of the frames. Variance component analysis (SAS, PROC VARCOMP) was used the obtain estimates of the inter-frame variance in AUC (V_AUC_) for rotation and no-rotation conditions. Variance estimates for the two conditions were compared based on an F-test. Statistical significance was determined based on α = 0.05.

## Authors' contributions

HML designed the study, participated in subject testing and drafted the manuscript.

DMR provided the expertise for the variogram analyses and participated in writing the manuscript.

JRF wrote software for data analysis, participated in the human subjects testing and writing the manuscript

JW acted as a consultant for the interpretation of ultrasound findings and contributed to writing the manuscript

EEK acted as a consultant for the analysis and interpretation of elastography findings and contributed to writing the manuscript

DST recruited and coordinated human subjects testing and participated in manuscript preparation

NAB participated in the human subjects testing and performed ultrasound data analyses

GJB participated in the design of the study, performed the statistical analysis and participated in writing the manuscript.

MHK participated in the study design, performed the biopsy excisions and participated in writing the manuscript

All authors read and approved the final manuscript.

## Supplementary Material

Additional File 1 3-d animated rendition of collagenous sheets corresponding to ultrasound image shown in Figure 2C Click here for file
